# River metrics by the public, for the public

**DOI:** 10.1371/journal.pone.0214986

**Published:** 2019-05-08

**Authors:** Matthew A. Weber, Paul L. Ringold

**Affiliations:** 1 University of Maryland, Center for Environmental Science, Solomons, Maryland, United States of America; 2 U.S. Environmental Protection Agency, Office of Research and Development, Western Ecology Division, Corvallis, Oregon, United States of America; Universitat de Barcelona, SPAIN

## Abstract

Managing rivers in society’s best interest requires data on river condition. However, the complexity of river ecosystems, combined with finite budgets for river monitoring and modeling, mean difficult choices are necessary regarding what information will be available. Typically, decisions of "what to measure" are left to natural scientists. However, knowledge of public appetite for different types of information helps ensure river data is useful to society. We investigated public interest in rivers directly, engaging nearly one hundred urban and rural participants in a combination of focus groups and semi-structured interviews. Drawing on concepts of "final" ecosystem services developed in environmental economics, we moved discussions past commonly mentioned stressors, such as pollution, to actual river features important in and of themselves. Participant feedback reflected extensive thought on river issues, in contrast to a stereotype that the public is ambivalent about environmental conditions. Interests were also broad, encompassing water quality and quantity, fish and wildlife, vegetation, and human features. Results show consolidation around relatively few themes despite diverse sociodemographics. Themes were interpreted into distilled, specific metrics to make public feedback as useful as possible for water resources monitoring, modeling, and management. Our research provides detailed, methodically generated hypotheses regarding river themes and metrics of public interest that should be considered as part of the tradeoffs inherent in river monitoring design. Results compared reasonably well to river attributes emphasized in river restoration environmental valuation reviews, with some differences. Future research could test our hypotheses with large-sample surveys.

## Introduction

River ecosystems are multi-attribute public goods. Decisions about what to manage and report on are often left up to biophysical specialists. For example, water resources journals are replete with technical monitoring guidance and insights about attributes to measure. However, the goal of managing public goods necessary involves decisions about public interests in these resources. Our premise is that decisions about how to describe rivers to the public and therefore guide management should be informed by increased knowledge of the specific elements of public interests.

Our approach in this article is to collect preference data by asking members of the general public what is important to them about rivers, via established qualitative research techniques used in such fields as sociology, anthropology, and marketing. This helps document public perspectives so that they can be considered in the difficult choices involved in targeting measurements used to represent river condition. This increases communications potential since the public is more likely to be interested in tailored, accessible information. Likewise, management decisions in the public interest are more likely when a foundation of knowledge is available regarding what is important to a constituency. For example, public preferences could inform priorities for restoration budgets given the broad range of potential outcomes restoration programs might deliver.

We wish to be clear that technical counsel is not the input we sought from our research participants, nor do we believe the public would (generally) be qualified in this regard. Instead, we asked the public to comment, in lay terms, on measurable features ultimately important to them about rivers and streams. Given information on such “goalposts,” technical approaches to measure, model, or predict the desired information could be formulated by natural scientists. If matching information is found to already be available, public feedback helps tailor that information for public audiences. Natural scientists have expressed interest in incorporating public interest in stream monitoring design and monitoring in general [1–3].

Our approach is aligned with the “final” ecosystem goods and services conceptual model [4– 9], hereafter referred to as Final EGS. Whereas ecosystems are composed of multiple interconnected processes and functions, the Final EGS hypothesis is that this complexity can be reduced to a smaller subset of ecological attributes highly relevant to people. These attributes rely on myriad underlying or "intermediate" ecological factors, yet provide a way of focusing natural science and social science attention on key ecological outcomes. People do not necessarily need to understand ecological processes in order to describe the final ecological outcomes directly relevant to them. These attributes are then in turn the currency of interdisciplinary work. Social science efforts of multi-objective planning, decision science, cost-effectiveness analysis, nonmarket valuation, and benefit-cost analysis all rely on articulated attributes; these tools can be deployed to assist in managing public goods for broad public interests, rather than special interest groups.

Piecemeal insight on publicly-valued river metrics is available from multiple sources. For example, nonmarket valuation case studies featuring rivers tend to carefully define metrics and changes to be valued. For an example of a US national scope study, see [10], and for local examples see studies included in the review [11]. However, despite this literature, it can be difficult to gauge the public relevance of metrics used, since an explanation of how metrics were selected and validated, from all the possibilities, is typically incomplete. For example, [10] simply use water quality metrics derived from early US Environmental Protection Agency National Water Quality Inventory metrics [12]. This is emblematic of the nonmarket valuation literature: instead of metrics emerging from exploratory empirical social science study, metrics are most commonly derived from needs of the funder, researcher-driven objectives for the experiment, or information that happens to be available. Thus, although metrics from any given study may seem sensible, it remains unknown which metrics would be the most relevant for the population surveyed. There may be important metrics left out, or people may attempt to extrapolate given metrics to those more relevant to them [7].

Certainly, many nonmarket valuation studies do rigorously test metrics, even taking pains to modify attributes to improve the experiment based on qualitative research findings, e.g. [13, 14]. However, even with such high standards, the number of variables that can be included in any single experiment is limited by human cognition [15]. Furthermore, the manner in which attributes are validated through pre-testing is by no means standardized [16]. Thus, despite availability of case-study literature carefully defining river metrics, there remains a need for a broad, exploratory research.

We are aware of only scant prior research investigating public interest across the spectrum of river attributes. An early study by [17] presented a small number of research participants with existing US Environmental Protection Agency monitoring data for rivers and other ecosystems, and used their input to propose ways of repackaging existing data. The study [18] is the first of which we are aware attempting to build the breadth of publicly valued river attributes *de novo*, i.e. considering what data to collect without constraint. They convened an expert workshop of natural and social scientists to hypothesize a list of Final EGS for rivers, for a variety of beneficiaries such as industrial or recreational users, and these results also appear in [19]. A subset of these Final EGS were described in more detail for a subset of beneficiaries in [6]. Later, [20] documented Final EGS as defined by the general public, engaging urban residents of southern Arizona.

Our goal in this paper is to continue the example of these studies, providing broad insight and hypotheses regarding river themes important to the respondent population, unconstrained by the agenda of any specific river management project. Furthermore, each theme of public interest is interpreted into one or more specific river metrics. These metrics extend public input to foresee and assist with the decisions needed to collect data at an actual field site, to make the results as relevant as possible for water resource monitoring, modeling, and management. Thus, our results create a bridge between social science and natural science arenas within the water resources discipline. This article continues with the tested empirical techniques of [20], extending the research into the Pacific Northwest, and for the first time including both urban and rural participants. We synthesize findings across the two empirical studies in the Overall discussion, and also provide a broad comparison with the nonmarket valuation literature on river restoration.

## Methods

The study setting is the Willamette watershed in western Oregon. Approximately 2.5 million people reside in the watershed, comprising the bulk (65%) of Oregon’s population. The Willamette River drains 2.97 million hectares between the Coast Range and Cascade Mountains before meeting the Columbia River northwest of Portland. The valley is known for agricultural production of grass seed, fruits, vegetables, and grains, as well as significant industrial and public timberlands. The region is noted for temperate, wet winters and dry summers. The Willamette River is ranked 19^th^ in US rivers by annual flow, averaging 1.06 thousand cubic meters per second [21].

Focus groups and semi-structured interviews were utilized for field data collection. These in-depth techniques are ideal to explore and document river attributes important to the sampled residents. Back-and-forth opportunities afforded by qualitative research methods were indispensable for ensuring input on specific, measurable, attributes important in and of themselves (i.e., Final EGS, rather than proxies). The US Environmental Protection Agency Human Subjects Research Review Office reviewed the study and approved it (exempt) with reference number C10-013CS. To recruit participants, we utilized a purposive sampling strategy which allowed us to gather perspectives from typical residents, but also explore similarities and differences between population segments. We used five mutually exclusive segments as follows: urban low-income; urban recreationalist (and not low-income); urban non-recreationalist (and not low-income); rural farmers; and rural non-farmers. Any given person could qualify for one and only one of these segments. Two geographic areas of recruitment within the watershed were designated for each of the five population segments to keep the segments from reflecting solely a single area. Urban recruits came from both Portland and Corvallis, and rural recruits came from both the Linn-Benton and the Clackamas-Marion county areas.

The rationale for separating urban from rural groups was to investigate whether the so-called “urban-rural divide” would manifest in divergent preferred river attributes. Within the urban population, recreationalists were separated in the event they might have more specific river preferences due to their activities, or would tend to overwhelm input from others. We separated low-income persons in order to examine whether there were any particular river preferences particular to disadvantaged persons. Urban non-recreationalists were separated since it is difficult to hypothesize what river attributes this group might prefer since they have no apparent special engagement with the resource. In considering rural residents, our anecdotal information was that farmers felt over-regulated by environmental policies designed to protect rivers, thus they might have a different perspective. Rural farmers were separated from non-farmers as a way of distinguishing two recognizable but separate segments of rural life, since most rural residents of Oregon do not partake in or gain significant income from farming (personal communication, Bruce Weber, Oregon State University Rural Studies Program).

Population segment definitions were as follows. The low-income criterion was defined as 185% or less of the US Census poverty threshold by size of household [22, 23]. Recreationalists were defined as persons being on or near bodies of fresh water for the purposes of recreation on average once per month. Rural was defined using the US Census definition of residence in a community of 2,500 persons or less in open country. Farmers were defined as those self-identifying as receiving significant household income from farming, with non-farmers self-identifying as not receiving such income. Table 1 summarizes the population segments, screening criteria, and recruiting locations.

**Table 1:** Population segment definitions.

Segment	Criteria	Area of Residence
Urban Low-Income	Residing in Portland or Corvallis; Below 185% of low-income threshold for household size based on US Census	Portland or Corvallis
Urban Recreationalist, (and not Low-Income)	Residing in Portland or Corvallis; Above 185% of low-income threshold for household size based on US Census; recreation on or near water bodies more than once per month, on average	Portland or Corvallis
Urban Non-Recreationalist, (and not Low-Income)	Residing in Portland or Corvallis; Above 185% of low-income threshold for household size based on US Census; recreation on or near water bodies no more than once per month, on average	Portland or Corvallis
Rural Non-Farming	Reside in a community of 2,500 persons or less in open country; no significant household income from farming	Linn, Benton, Clackamas, or Marion Counties
Rural Farming	Reside in a community of 2,500 persons or less in open country; no significant household income from farming	Linn, Benton, Clackamas, or Marion Counties

A marketing firm was contracted to recruit the participants, relying mainly on cold calls from a random sample phone list for each defined region. Internet notices and flyers in local libraries were used to supplement cold call recruiting. Farmer groups proved especially difficult to recruit; only for this population segment were a few referrals permitted (for other farmers). To reduce self-selection of those with a particular interest in river and stream issues, the recruiter offered an incentive payment of $75 to attendees. A screening script was used in which the recruiter was not permitted to describe the topic of the meeting as being rivers and streams. Our rigorous recruiting measures are a strength of the study, contrasting with “open-door” meetings, or inviting predefined “engaged” stakeholders. The intention was that input received would be more likely to reflect broad-based issues within diverse population segments rather than being overtaken by potentially narrow interests. To further diversify input, there were at least two groups per segment and at least one for each geographic area. All groups were moderated by the lead author for consistency, and convened in local library branches, to offer comfortable and neutral surroundings for participants.

An open-ended script comprised of relatively few questions structured the meetings (S1 Appendix). The script follows a common “funnel” design of warm-up, intermediate, and main questions [24]. The questions asked participants about rivers and streams in western Oregon in general, as well as about the Willamette River in particular. Focus groups were approximately two hours in length. Brief background as well as photo visual aids for the Willamette River were provided halfway through the meetings (S2 and S3 Appendices). Photos were selected to display a variety of conditions on the Willamette River mainstem, to seed discussion. Photo A is by Miles Hochstein (https://portlandground.net), used with permission. Photos B, C, D, and F were from US Environmental Protection Agency stream monitoring files. Photo E is from [25], used with permission. We carefully selected background information and photos to offer various portraits of the resource, yet any choice of information provided necessarily incurs a bias. We believed the advantages of visual and text information to seed respondent reactions would outweigh the disadvantages, since many participants might not be closely familiar with the resource. A worthwhile avenue for future work would be to repeat the study with different background material and photos.

Focus groups were tape-recorded and transcribed, providing a tremendous dataset of over 300,000 words for analysis. Participant input was distilled through an iterative process of reading transcripts to identify emergent themes corresponding with measurable attributes of rivers and streams. In all, there were more than 2,500 code occurrences for measurable features. We report code frequencies to convey a sense of the relative importance of different themes, although there is debate regarding this practice [26] (p.185). We employ qualitative techniques for their hypothesis generating potential [27, 28]; our code count results are not meant to imply an ability to statistically test hypotheses or generalize findings to the broader population. Like many qualitative studies, we provide a depth of input with an intensively studied small sample, and investigate variation by deliberately engaging diverse participants. We view our study as part of a vanguard of exploratory research on empirically derived river metrics, to provide a reference for other research deriving or utilizing river metrics, including large-sample quantitative approaches to verify public priorities suggested by our code count frequencies. For example, choice experiments techniques can be used to rate the relative importance of different attributes, rather than quantify the value of changes in given attributes [29, 30].

To systematically code transcripts for frequency analysis, turns of talk were used as a unit of text [31] (p.105). A block of text in which a participant was briefly interrupted, but then continued speaking was treated as a single unit of text for that speaker. A given theme could be coded only once for each unit, but could be coded multiple times if the speaker returned to a topic repeatedly throughout a transcript. Many separate themes could be coded within a given paragraph, for example the phrase “endangered fish” would require citing an occurrence for both the fish theme and the endangered species theme. Rare measurement themes raised only by one person or arising in only one focus group are not reported. Certain recreational activities mentioned by participants clearly imply river measurements; such cases are noted in the results. To provide additional detail in how different measurement-oriented themes were raised, several emergent *context* codes were tracked (as opposed to measurement codes). These context codes are not tabulated to save space, but are described in the text, with full results available from the authors upon request. Coding and analysis were facilitated by The Ethnograph (v6) software [32].

After initial analysis of focus groups, themes of public interest were interpreted into specific metrics of sufficient detail to guide river data collection. Subsequently, an additional phase of data collection we refer to as validation interviews occurred to apply a form of "member-checking" to the metrics [33, 34] (p.201). These new participants were provided with a summary sheet of river measurements for their reaction. In addition to their open-ended reactions, interviewees were asked to highlight five measurements that they would emphasize, as well as five measurements they would drop if resources were constrained, to assist in identifying priorities. Participants were again recruited with the assistance of a marketing firm with the same protocol as with focus groups. The semi-structured validation interviews were approximately one hour, conducted by telephone. Interviews were transcribed and coded using the same techniques as for focus groups.

## Results and discussion

**Fig 1:**
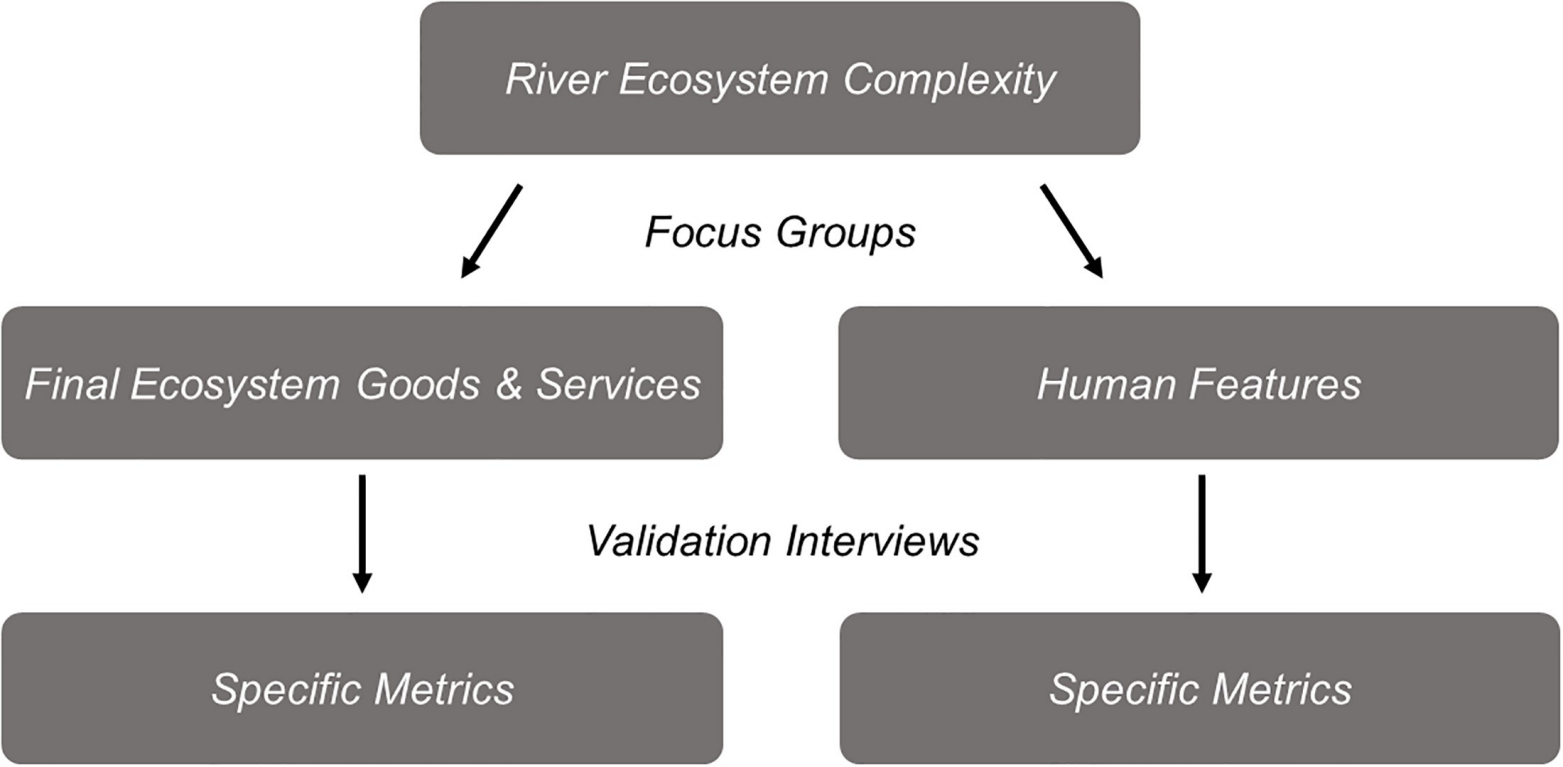
Methodology flow.

Sociodemographics for all population segments are shown in Table 2, illustrating participant diversity. Final EGS themes (Table 3) were organized in a structure as follows. The first branch of the hierarchy separates inert and live features, with Water and Channel for the former, and Vegetation, Fish and Wildlife, and overarching Biota issues for the latter. Water is further split into subcategories of Quality versus Quantity. These categories reflect the breadth of participant input. For example, the Biota category was needed due to the emergence of themes such as nuisance species and native species that apply across different types of wildlife and vegetation. No attempt was made to limit participants to input on ecological, or ‘natural’ features, and we also coded input on numerous anthropogenic river attributes. To distinguish these, results are divided into Final EGS versus Human features. This helps isolate the ecological component of rivers important to people as opposed to onsite human modifications serving as either amenities or disamenities. The methodology flow is represented in Fig 1.

**Table 2:** Participant sociodemographics^1^.

Segment	Urban Low-Income	Urban Rec.	Urban Non-rec.	Rural Non-Farming	Rural farming	TOTAL
# Focus Groups	2	3	2	3	2	12
# Validation Interviews	2	2	10	2	2	18
Total # Participants	19	20	27	16	17	99
Source						
Cold Call	10	16	21	15	13	75
Internet	7	3	6	0	0	16
Flyer	2	1	0	1	0	4
Referral	0	0	0	0	4	4
Gender						
Male	10	9	13	9	11	52
Female	9	11	14	7	6	47
Age Group						
18–29	5	1	3	0	1	10
30–39	2	1	1	1	0	5
40–49	5	7	6	0	1	19
50–59	4	5	10	6	6	31
60+	3	6	7	8	8	32
Household Income						
<20k	13	0	0	1	1	15
20k to 39k	6	2	5	2	3	18
40 to 59k	0	4	7	7	4	22
60 to 79k	0	8	7	1	2	18
80 to 99k	0	2	0	1	0	3
100+k	0	2	8	1	4	15
Employment						
Homemaker	0	1	1	0	1	3
Student	1	0	1	0	0	2
Other Employment	10	12	21	10	1	54
Unemployed	2	3	1	0	1	7
Retired	5	4	2	5	0	16
Disabled	1	0	0	0	0	1
Rural farming	0	0	0	0	14	14
Yrs in present location						
0 to 9	4	2	3	1	2	12
10 to 19	7	6	8	4	1	26
20 to 29	4	4	9	1	4	22
30 to 39	3	3	5	3	3	17
40 to 49	0	3	2	1	0	6
50 to 59	1	1	0	4	4	10
60 or more	0	1	0	1	2	4
Yrs in Pacific Northwest						
0 to 9	3	0	1	0	0	4
10 to 19	4	6	3	2	1	16
20 to 29	4	2	6	1	2	15
30 to 39	3	1	6	3	2	15
40 to 49	3	7	5	1	2	18
50 to 59	2	1	5	5	7	20
60 or more	0	3	1	3	2	9
Number of Water-Related Recreational Trips per yr						
cumulative	1178	1533	121	783	531.50	4146.5
count	19	20	27	15	16	97
avg	62.0	76.7	4.5	52.2	33.2	42.7
Residence						
Portland	9	9	13	0	0	31
Corvallis	10	11	14	0	0	35
Rural Linn or Benton Co.	0	0	0	8	10	18
Rural Marion or Clackamas Co.	0	0	0	8	7	15

^1^: Breakdowns may not sum to segment totals due to item nonresponse, or individual response varying from household response. For example, a respondent may be from a rural farming household, but have a different individual vocation, such as homemaker.

**Table 3:** Themes, metrics, and segment relative frequencies for river final ecosystem goods and services, and human features. Each segment sums to 100%.

Category	Participant Theme	Theme Metric	Metric Explanation	Urban Low-Income (total code count = 435)	Urban Rec. (total code count = 590)	Urban Nonrec. (total code count = 630)	Rural Non-Farming (total code count = 591)	Rural Farming (total code count = 297)
Water Quality	Concern about safe recreational contact with river water	WQL1	Probability of water-borne illness from partial body contact and full body contact with river water	3%	5%	5%	6%	3%
	Concern about water supply health risks	WQL2	Probability of water-borne illness from drinking tap water associated with river water	3%	4%	3%	3%	2%
	Interest in health risks of drinking water directly from the river	WQL3	Probability of water-borne illness from drinking river water	2%	2%	1%	2%	3%
	Appreciation of water clarity	WQL4	Clarity of water, depth of visibility	3%	1%	1%	3%	1%
	Appreciation of visible flow	WQL5	Whether or not there is perceptible flow	2%	0%	1%	2%	2%
	Interest in water temperature	WQL6	Temperature of water in Deg. F and Deg. C.	3%	0%	1%	1%	1%
	Interest in water views	WQL7	Percent of water surface visible in summer, from 100-year floodplain boundary	1%	1%	1%	0%	1%
	Appreciation of the sound of flowing water	WQL8	Presence of the sound of flowing water	1%	0%	1%	0%	0%
	Interest in water taste	WQL9	Local tap water taste rating	1%	0%	0%*	0%*	0%*
Water Quantity	Concern about property damage from floods	WQN1	Annual probability of flooding inundating sensitive property	1%	2%	3%	5%	21%
	Interest in water supply quantity	WQN2	Minimum surface water flow, and minimum surface water volume per year	4%	4%	3%	2%	5%
	Interest in flooding as a natural phenomenon	WQN3	Width of predevelopment 100-yr floodplain as compared with current condition	2%	2%	1%	1%	0%*
	Interest in hydropower	WQN4	Currently used and unused hydropower potential	0%	1%	1%	0%	1%
Channel	Interest in navigation	C1	Minimum main channel depth and width, class of rapids, and presence of navigation hazards such as downed trees	6%	5%	4%	4%	9%
	Interest in swimming	C2	Whether minimum thalweg depth allows for swimming	2%	2%	1%	4%	4%
	Concern about erosion unrelated to property	C3	Degree of erosion and degree to which influenced by human activities	2%	2%	1%	1%	1%
	Interest in ease of access to water’s edge	C4	Ease of access from 100-year floodplain boundary to the edge of the water, including presence of sandy beach	3%	1%	1%	1%	0%
	Interest in camping areas	C5	Presence of flat, smooth areas suitable for tents during summer flow conditions	0%*	1%	0%	1%	1%
	Concern about swimming hazards	C6	Presence of swift currents, hydraulics, or submerged objects hazardous to swimmers	1%	1%	1%	1%	0%*
	Interest in woody debris	C7	Presence of large stream-carried woody debris	1%	0%	0%	0%	0%
	Interest in rocks and outcrops	C8	Description of rocks and presence of bedrock outcrops	1%	1%	0%	0%	0%
Biota	Appreciation of native species or dislike of invasive species	B1	Presence and abundance of invasive plants or wildlife	5%	10%	6%	4%	3%
	Dislike of nuisance species	B2	Presence and abundance of plants or wildlife known to harm humans or damage property	3%	1%	2%	3%	3%
	Concern for sensitive species	B3	Presence and condition (such as mutations) of species sensitive to environmental conditions such as frogs or amphibians	1%	2%	3%	1%	0%*
	Concern for endangered species	B4	List of plant or wildlife species present that are in danger of extinction	1%	1%	2%	1%	1%
	Interest in plant and/or wildlife diversity	B5	Total number of different types of appreciated fish and wildlife species (aggregate appreciated Vegetation and Fish and Wildlife themes)	0%	0%	1%	0%*	0%*
Aquatic Wildlife	Appreciation of fishing opportunities or reference to common game fish	AW1	Presence and abundance of game fish species, including salmonids	6%	7%	10%	17%	12%
	Appreciation of the presence of fish (not necessarily game fish)	AW2	Presence and abundance of all fish species	5%	6%	5%	5%	4%
	Concern of safe fish consumption	AW3	Edibility (toxicity) of game fish	1%	3%	2%	4%	1%
	Appreciation of aquatic life other than fish	AW4	Presence of aquatic wildlife besides fish, such as amphibians, crayfish, or microorganisms	1%	2%	3%	1%	1%
	Appreciation of fish taste	AW5	Taste rating of game fish	0%*	0%	0%*	1%	0%*
Land Wildlife	Appreciation of birds	LW1	Presence and abundance of bird species, esp. birds-of-prey such as ospreys/eagles/hawks, and large birds such as herons/ducks/geese	4%	4%	3%	3%	4%
	Appreciation of mammals	LW2	Presence and abundance of mammal species, esp. larger mammals, including predators	4%	3%	4%	3%	1%
	Appreciation of reptiles	LW3	Presence and abundance of reptile species	0%*	1%	1%	1%	0%
	Appreciation of insects	LW4	Presence and abundance of insect species	1%	0%*	1%	0%*	0%*
Vegetation	Appreciation of large trees	V1	Presence and abundance of trees, including large trees	5%	4%	4%	4%	2%
	Appreciation of greenery or lush bank vegetation (other than trees)	V2	Presence and abundance of lush green vegetation including shrubs and grass (does not include large trees), and whether manicured	3%	1%	1%	1%	2%
	Dislike of algae	V3	Presence and abundance of algae	1%	1%	0%	1%	1%
	Appreciation of wildflowers	V4	Presence and abundance of wildflowers	0%	1%	1%	0%*	0%*
	Appreciation of edible plants	V5	List of plant species present that are edible	0%*	1%	1%	0%*	0%*
Human	Appreciation of recreational amenities	H1	Presence and extent of paved and unpaved trails, interpretive signage, boat ramps or other boating facilities, bathrooms, public transit to river areas. Presence of any developed campground facilities including handicapped facilities. Cost of access.	9%	6%	4%	3%	2%
	Dislike of garbage	H2	Presence and abundance of garbage	3%	3%	5%	4%	2%
	Interest in legal access	H3	Whether site and any nearby road access is on public or private land	0%	2%	3%	3%	1%
	Dislike of crowding or appreciation of people using the river	H4	Number of recreational users present	2%	3%	2%	2%	0%
	Interest in esthetics of human infrastructure	H5	Description of human infrastructure (besides recreational amenities) visible in summer from edge of river	3%	1%	2%	1%	1%
	Dislike of human-caused odor	H6	Presence and description of odor of human origin	2%	1%	1%	1%	2%
	Dislike of human-caused noise	H7	Presence and description of sound of human origin	1%	1%	2%	0%	1%
	Interest in land use context	H8	Description of land use within the 100-yr floodplain	0%*	0%	1%	0%	0%*
	Concern of crime	H9	Crime rate and crime description (other than littering or dumping)	0%	0%	0%*	0%	0%*

* = True zero; no code occurrences for this segment

**Fig 2:**
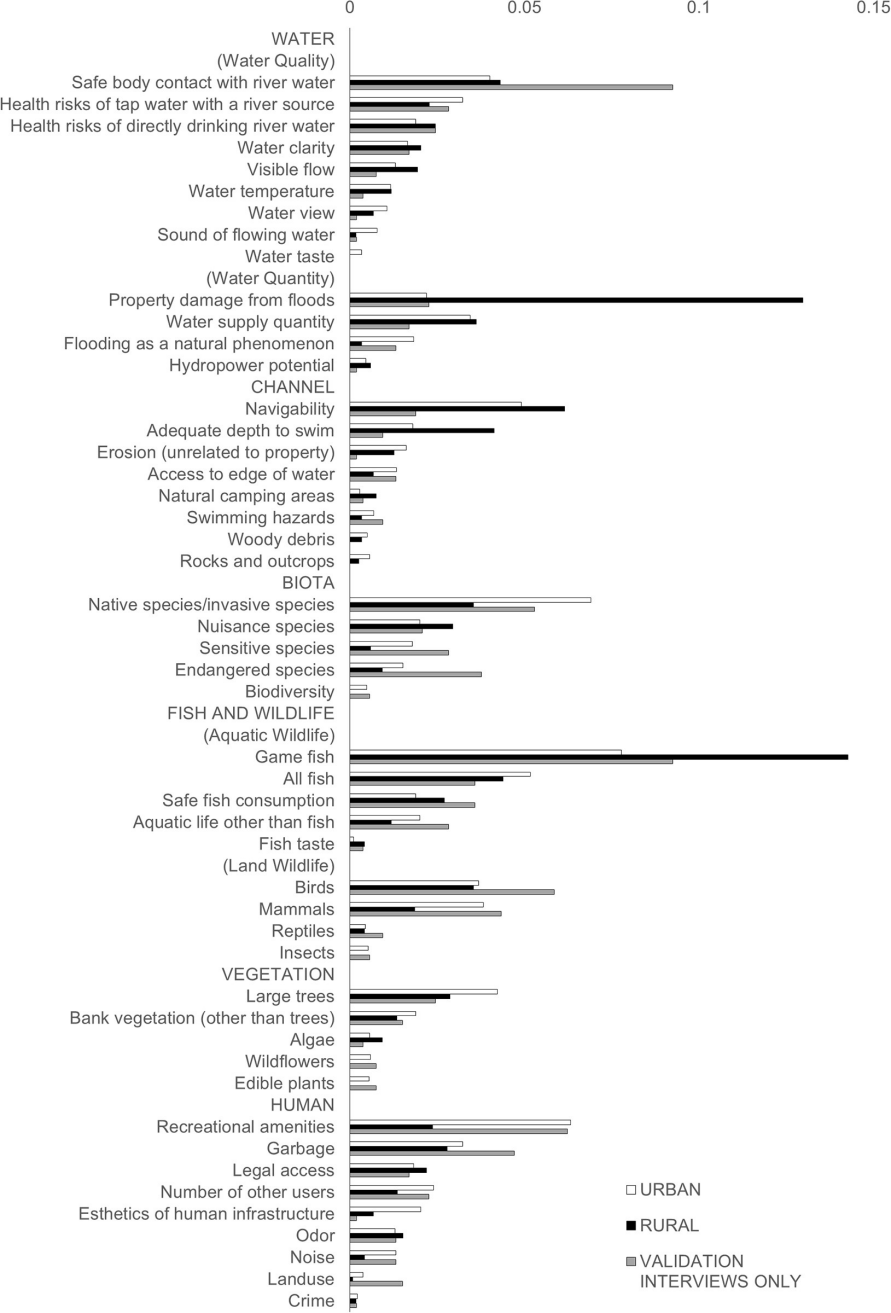
Theme frequency percentages; each population segment weighted equally within the urban or rural class. Validation interviews included in urban and rural results and also broken out as a separate series. Bars sum to 100% for each series.

**Fig 3:**
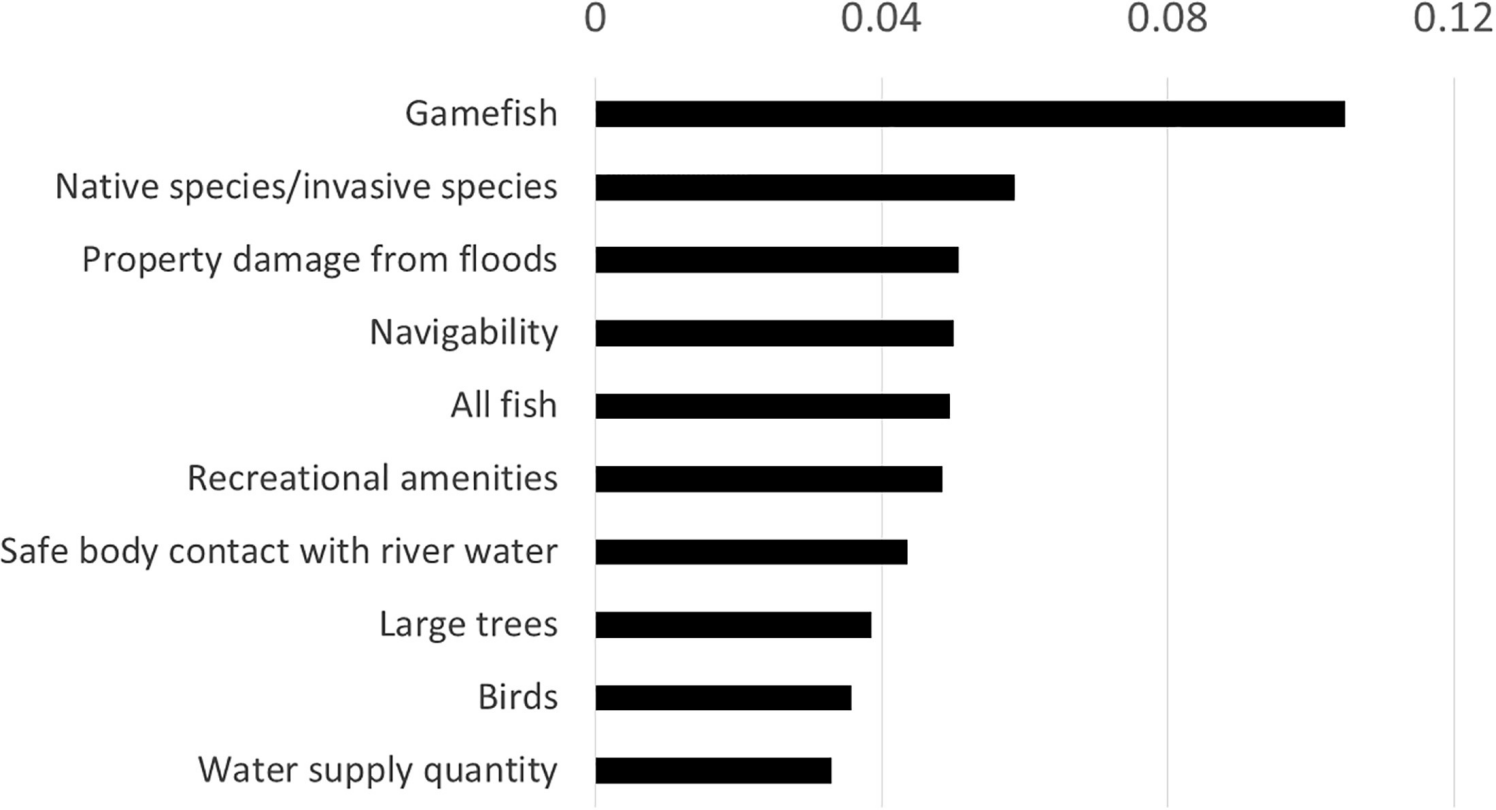
Top ten theme frequency percentages; all segments combined. These themes account for the majority of combined input (bars sum to 51%).

Code frequencies (Table 3) are normalized to within-segment percentages for each theme, for focus group and validation interview participants combined. Normalization puts data from each population segment on equal footing as compared with other segments. Measurement themes and corresponding metrics and shorthand are listed in descending frequency within category in Table 3. Frequencies for urban versus rural participants are graphed in Fig 2; validation interview feedback is included in the appropriate urban or rural segment, and also broken out as a separate series. Frequencies for the top ten themes shown in Fig 3. In the ensuing discussion, additional context is given for each theme, with attention to where population segments differed, as well as any relevant insights from validation interviews.

### Water

#### Water quality

We isolate nine distinct Final EGS themes and metrics associated with water quality. Foremost, participants were highly aware that rivers are a source of drinking water, and showed strong concern about water supply contamination. Despite sophisticated water treatment, concern remained for treatment system vulnerabilities (WQL2). There were also a number of comments pertaining to the safety of drinking water directly from the stream, tabulated as a separate metric (WQL3). A “drinkable” river water quality is the top tier in the classic water quality “ladder” used in economic valuation of water quality improvements such as US Environmental Protection Agency regulatory impact assessments [35], however achieving this tier has not been prominent in actual valuation studies.

Nearly equal to the combined interest in water supply quality found in WQL2 and WQL3, was concern about the safety of recreational contact, represented in WQL1. Nearly all of these comments reference swimming or submersion, with a few quotations each for boating, wading, or unspecified contact. Swimmable and boatable water quality levels are familiar attributes in the literature, also reflected in the classic water quality ladder [35, 36]. A majority of validation interviewees selected safety of recreational contact as one of their top five: far more votes than any other metric received.

There were several comments having to do with visible water quality. Water clarity was preferred, with many comments on esthetics (WQL4). Clarity as a desired surface water attribute is supported by many studies, e.g. [37, 38]. However, in our data it was apparent that clarity was also thought of as signaling other water quality concerns, such as the safety of the water supply or the safety of body contact; in other words, other Final EGS. In such cases the final outcome of interest was tallied instead of clarity.

A separate theme for visible flow was coded due to negative comments regarding “stagnant” rivers (WQL5). A final theme regarding visible water quality was appreciation of a view of water (WQL7), including waterfalls.

Remaining water quality themes have to do with perceptions other than health or visible quality concerns (WQL6, WQL7, WQL8, and WQL9). When temperature of river water was discussed (WQL6), this included recognition of cold water being a more natural water quality status. Notably, there are more than 3,000 US waterbodies listed as impaired due to temperature pollution [39].

#### Water quantity

The single most prevalent water theme was concern for property damage by flooding (WQN1), as easily seen in Fig 2. This was especially true of farmer participants, who described firsthand experience with high flows impacting their land alongside rivers, and perceived lack of ability to take erosion control measures. There were also numerous mentions of property damage by urban groups, yet it was elevated to such a degree for rural groups that we mark it as an urban/rural difference.

About 20% of flooding-related quotations did not involve property concerns. Instead, the emphasis was on flooding as a positive ecosystem feature or simply as a notable phenomenon. These comments led to our inclusion of a metric gauging the extent of flooding as compared to past conditions (WQN3). Farmers never described positive aspects of flooding, despite some non-farmers perceiving flooding as a boon to farmland productivity. There were numerous comments from non-farmers regarding “poor planning” of investing in infrastructure such as crops or buildings within a flood zone, usually in reaction to photos E and D. In contrast, farmers were more likely to view photo E as a situation out of the landowner’s control.

A separate theme within water quantity was oriented to water supply (WQN2). The necessity of water for municipal needs such as drinking water was most prevalent, closely followed by the need to supply agriculture, with industrial water needs last.

The last theme under water quantity reflects a dozen comments on the importance of rivers for supplying hydropower opportunities (WQN4). There were also anti-dam comments; upon follow-up from the moderator these were found to be connected to Final EGS themes such as fish, or flooding as a positive characteristic.

### Channel

The largest channel theme was suitability for navigation (C1), mainly in association with recreation. The context was participants expressing an interest in activities such as boating, rafting, canoeing, kayaking, or tubing, which implied an interest in navigability. There were less than a dozen quotations describing actual boating hazards, which tended to be from rural participants. About 25% of the C1 quotations mention the importance of rivers for commercial transport such as commodities.

Swimming was another frequently mentioned activity, voiced twice as often by rural as urban participants. Interest in swimming implies a measurement of sufficient channel depth (C2). A safety concern was hazardous currents for swimmers (C6). People also frequently described difficulty accessing the water’s edge: places with an easier natural access such as a beach-type shoreline were preferred for recreational activities (C4). The presence of areas suitable for camping was appreciated, such as flat ground on the riverbank (C5). Comments on developed campground facilities were coded under Human features.

There were numerous comments regarding erosion that were not related to property damage or water clarity concerns (C3). Reactions to photo E and F account for most occurrences of this theme. The context was usually unspecified. Some simply disliked the esthetic, others worried that erosion would be bad for the “health” of the river. Downcutting and reduced bank stability from human impacts is a well-known issue among river ecologists [40].

Germane to channel preferences were a number of comments regarding downed wood in the waterway. Due to the importance of woody debris for habitat purposes and river restoration efforts, public perception of downed wood has been a topic of survey research [41–43]. In general, our qualitative findings support previous results for Oregon, although our quotations are useful for understanding detailed perceptions. All of the positive comments regarding downed wood occurred after photos were distributed and directly referenced the woody debris on the shoreline in photo B. The context was that the wood signaled a natural condition, e.g. fish habitat. For the people noting downed wood as a negative characteristic, some thought that woody debris could impede fish passage, for others it represented a form of property damage, and two people wondered whether it indicated logging in the area (and would thus be unnatural). The code frequency of woody debris as a Final EGS and associated metric (C7) is reserved for when it was not a proxy for other attributes, but perceived as a natural condition.

Last in terms of channel characteristics, rocks and outcrops in association with rivers were of interest to several persons (C8).

### Biota

Several themes apply to both fauna and flora; thus the overarching category of Biota was required. The central finding was strong interest in native species. Native species was coded not just for the term “native” but also terms or phrases such as “indigenous,” or wildlife “supposed to be there.” There was an even larger number of comments disliking invasive species, with a worry of them “taking over” native species. In addition, there were numerous references to endangered or extinct species, by definition a reference to native species. Finally, there were several occasions of disliking nonnative species regardless of whether they were invasive or not. All these comments were distilled into two themes and metrics, one theme on invasive species concerns and native species appreciation (B1), and one theme for endangered species (B4).

The context of native species appreciation comments was about one third generalized, one third in regards to fish in particular, and one third split across various plants and wildlife. For invasive species, most comments pertained to invasive wildlife, mainly fish, followed closely by invasive plant references, followed by comments unspecific to either animals or plants. Endangered or extinct species were mentioned by name infrequently. The few mentioned were salmon, birds, and mammals. More than half of the references to endangered or extinct species were made by validation interviewees. Furthermore, all interviewees were asked whether they were more interested in information on wildlife that could be hunted or fished, or endangered wildlife (S1 Appendix): twice as many chose the latter. Endangered species issues are sometimes considered controversial, and it is possible that the individual interview data collection phase allowed participants more freedom to voice their opinion [44]. It should also be noted that the background information (S2 Appendix) mentions nonnative fish and endangered fish. This likely influenced the frequency of these codes.

There was a recurrent but minority view to the effect that native, invasive, and endangered species concerns were not that important. The frequency of this view, tracked as a context code, was at 10% of the corresponding themes. These minority sentiments declared historical status was not necessarily preferred today; it was a lost cause; and "survival of the fittest" regardless of which species arrived first.

Overall public support for protecting endangered species has been documented by other authors relying on survey results [45, 46]. Public affinity for native species in general has also been documented elsewhere [47, 48]. Studies investigating invasive species preferences have shown mixed opinions [49–51], whereas our results show only a few comments dissenting from an anti-invasive stance.

As if to balance the affinity for biota, there was also a theme for biota that was a nuisance or dangerous to people or human property, or that depleted game herds (B2). Most of the nuisance comments referenced wildlife, mainly predators such as wolves or cougars, followed by birds such as seagulls and geese that were annoying or were perceived as leaving large amounts of waste, followed by mosquitoes. There were only about a dozen nuisance plant comments. Overall, the sentiment on nuisance species was not that they should be eradicated, but rather: (1) they should be kept away from areas where they could harm people, (2) populations should be kept under control, or (3) one may simply wish to know what conditions to expect when considering recreation in a particular area.

A metric for sensitive species was created due to numerous concerns about the physical condition of animals or plants (B3). Almost all of these references were in regards to pollution-induced mutations in fish or amphibians such as frogs. In keeping with our coding protocol, such references were cross-coded, that is, concern about fish mutations would appear in the sensitive species theme, as well as the fish theme.

Interest in biodiversity is represented, but with a very small number of quotations (B5). The context of the comments seems to be related more to species diversity than genetic diversity, thus metric B5 represents the sum of appreciated species from Vegetation, and Fish and Wildlife categories. However, the topic was not prevalent overall, and further research is warranted on this complicated issue. Other authors have found that the public in general supports protecting biodiversity when the question is posed directly in both survey and focus group formats [52, 53].

### Fish and wildlife

#### Aquatic wildlife

Fish was by far the largest category for any type of aquatic life. Within this broad topic, game fish was the largest Final EGS metric (AW1), with a conspicuous code frequency (Figs 2 and 3). Most of the quotations were general references to the activity of fishing, implying an interest in game fish. Approximately half of these comments referred to salmon in particular. Salmon were also frequently mentioned in contexts *besides* fishing, indicating interest in salmon as a charismatic species, although we include such comments within the game fish Final EGS. Within game fish, there were also comments on hatchery fish. Rural participants tended to describe their preference for natives but their support for hatcheries in providing fishing opportunities. Urban participants tended to see hatchery fish as unnatural and suspected they harmed the natural ecosystem.

There was significant concern regarding safe consumption of fish (AW3). There were several references to varying fish taste (AW5).

A separate fish theme was made for comments mentioning fish that were not game fish, or generalized references to fish that lacked a clear fishing or fish consumption motivation. The "All fish" metric (AW2) was relatively large even at half the frequency of game fish, ranking second within aquatic life.

Various aquatic life besides fish was also mentioned, but to a much lesser degree, necessitating the relatively broad metric AW4. There were several mentions of unspecific aquatic life, but since these could not be associated with a specific metric, they were not tabulated.

#### Land wildlife

There were numerous vague references to “animals” or “wildlife,” or even just “habitat.” While this displays interest in wildlife, just as vague references to aquatic life were not tabulated, neither were these. Only when the moderator was able to probe participants for something more specific could input guiding a specific measurement be captured.

The most frequently cited specific form of land wildlife was birds (LW1). The largest specific subtheme was birds of prey, such as eagles and ospreys. References to other large birds such as herons, ducks, and geese also occurred but less often. The remainder, i.e. most of the bird codes, were unspecific, with only a few bird types mentioned.

Discussed nearly as frequently as birds were mammals (LW2). Interest in insects (LW3) and miscellaneous other forms of land wildlife (LW4) were minor themes.

When people were specific, large wildlife was more prevalent, as seen in the fish, bird, and mammal categories. There are at least three explanations for this. One is that larger wildlife is more visible and thus more charismatic. Indeed, the endangered species treated in nonmarket valuation studies tend to be easily visible and well-known types of animals [45]. Second, larger wildlife is more useful to people as a source of game. There were less than a dozen occasions of people mentioning hunting; however, it is possible that the association is subconscious. A final explanation is that larger species are often higher on food chain and thus represent keystone species, representing a healthy ecosystem overall. For mammals, we tracked interest in predators as a context code, which applied to several people. This included discussion about how predators keep ecosystems balanced. The perspective of predators playing a key role is well-supported in ecology [54].

### Vegetation

As with wildlife, there were many references to “plants” or “vegetation” with no further specifics, thus these vague mentions are not tabulated here. Only when the moderator succeeded in eliciting more detailed feedback could a measurable Final EGS be tallied. The most prevalent specific vegetation category was trees (V1). About a third of these quotations simply expressed liking trees. Positive impact on home value with increasing canopy vegetation has been documented in the area [55, 56]. About a fifth of the comments expressed a dislike of logging. The Pacific Northwest is relatively famous within the US for environmental controversy surrounding logging and clear-cutting [57], with a debate including but not limited to maintaining habitat for the endangered Northern Spotted Owl. There were a dozen occasions of trees mentioned as important buffer vegetation along streams. There were several mentions of the importance of trees shading streams for ecological reasons, several preferences for big, old trees, and a handful of comments appreciating the shade trees provide for people. Notably only ten tree comments mentioned specific tree types.

The second theme under vegetation, bank vegetation (V2), captures diverse comments. Appreciation of the greenery or lush vegetation along rivers was the most common (typically referencing natural vegetation, but also manicured), followed by citing the importance of riparian vegetation in providing a buffer, and handful of mentions of naturally occurring grasses. Relatively small themes were dislike of algae (V3), appreciation of wildflowers (V4), and interest in edible plants (V5).

### Human features

Many of the measurable features of rivers and streams that people described were clearly human controlled rather than ecological. Dislike of pollution permeated participant feedback, however, pollution is a stressor; thus our follow-up questions were designed to learn what ecological characteristics people worried about being damaged by pollution. Such follow-up interactions account for many of the Final EGS code occurrences inventoried in this article. Although we do not tabulate pollution as a theme, we did track several pollution context codes. Industrial, municipal, and agricultural pollution were all common, with agricultural pollution being somewhat less cited.

Recreational amenities were the most frequent measurable human feature of interest (H1). Activities of hiking, walking, or biking were taken to imply an interest in trails. Direct and implied interest in trails was the largest subtheme, constituting half of the amenity quotations, coming almost entirely from low-income and recreationalist segments. About two-thirds of the trail-related comments imply unpaved surfaces, e.g. references to hiking, while the remainder imply paved surfaces, e.g. urban “riverwalk” areas and bike paths. The second half of the recreational amenity quotations were split among several subthemes, also listed under the H1 metric. There were a large number of negative comments regarding trash (H2). Several people described carrying out other peoples’ garbage.

There was strong interest in legal access to rivers (H3). Most comments had the context of there not being enough access. However there were nearly as many comments simply interested in knowing where access was legal, expressing a need to balance access with private landholders, and a need to balance access with over-usage.

There were many references to humans themselves. These comments included both positive and negative statements (H4), with about twice as many of the latter. Urban participants in particular were concerned about over-crowding or desiring isolation at recreational sites. Crowding is a long-running theme in the recreation literature [58]. Comments in support of other users were in the spirit of wanting to keep opportunities open for others to connect with rivers. Several of these were associated with the easy access portrayed in photo A. There were also a few comments regarding crime (H9).

Human infrastructure besides recreational amenities was important. Nearly all such comments were in reaction to the photos distributed halfway through the focus groups, especially photo D, which shows a multifamily complex in close proximity to the Willamette River. Most comments viewed human infrastructure as a stressor, i.e. representing active or potential pollution. The prevalent context was that the buildings were “too close,” as well as suspicion of what pollution the culvert in the foreground could be carrying. Comments with a clearly esthetic context, such as “ugly” buildings were tabulated under an esthetics metric (H5). Also represented in this metric were comments that human-altered rivers could be attractive; about a dozen people had positive esthetic reactions to the city scene in photo A.

The themes of unnatural odor and unnatural noise (e.g. jet skis) also emerged as themes, for which we created metrics H6 and H7, respectively.

The metric for Land Use (H8) has a low code count, but is unique in that it emerged during the validation interview phase. As interviewees looked over the provisional list of metrics, and imagined actual stream information applying to different sites, several people desired land use information as a landscape level to contextualize stream data.

### Overall discussion

In broad strokes, several things stand out in the results. First, people discussed topics far outside of the river channel, ranging across vegetation, wildlife, even human features. Transcript narratives are also more sophisticated than might have been supposed, showing evidence of extensive thought on river issues, in contrast to a stereotype that the public is ambivalent about environmental conditions. At the same time, feedback was often generalized. Trees, fish, and birds were highly prevalent themes, yet people rarely mentioned specific types. We constrain metrics when we have sufficient evidence to do so, e.g. for birds we focus on birds of prey and other large birds (LW1). Whereas species-level disaggregation was not typically important for the metrics, other distinctions did matter. For example, interest in native species and nuisance species was relatively high. Metrics for endangered species and biodiversity also appear, but at low levels, despite being common topics in the ecological literature. In the case of endangered species, people may be reluctant to take a stand in a focus group on something perceived to be controversial; most of the endangered species code frequency occurred during individual validation interviews rather than group meetings.

The data collection was carefully designed to examine differences between segments, especially urban versus rural interest in rivers. We were prepared to record entirely different metrics and themes for different segments, but on the whole this was not necessary. Surprisingly, the main outcome from comparing results across segments was the *similarity* of Final EGS. That said, there was evidence of differences in attitudes–for example, a recurring rural complaint was the phenomenon of combined sewer overflows from cities into rivers, while agriculture faced unnecessarily strict runoff regulations. Yet despite attitudinal differences, in only a few instances were notable variations found in Final EGS. Namely, there was higher rural interest in flood property damage, game fish, and swimming. Urban interest was higher in how flooding has changed over time, mammals, invasive/native species, sensitive species, recreational amenities, and esthetics of human developments along rivers. There were a few additional themes with exclusively urban participation, but all sparse in frequency.

Overall, there was a remarkable coalescence of feedback. Similar river measurements repeatedly arose across ninety-nine diverse participants, and the thirty distinct focus groups and validation interview sessions we conducted. We only excluded measurement themes mentioned by only one person or one group–even so, we generated just 49 metrics from essentially unlimited possibilities. We also tracked the frequency of interest in a given theme relative to other themes, yielding evidence of further prioritization: the top ten themes account for over 50% of theme frequency (Fig 3). We provide supporting evidence for familiar attributes in the social science literature regarding appreciated ecosystem features, such as safe water contact, water clarity, fish, trees, and mammals. However, there were also a few surprises. There was a remarkable amount of concern regarding the safety of water supplies, despite sophisticated treatment and testing infrastructure. In a similar vein, there was interest in whether river water would be safe to drink, something we would not have thought many people would consider. A large number of participants had interest in boating or swimming, activities well represented even by the non-recreationalist segment, leading to high frequencies for navigation and adequate swimming depth metrics. Interest in native species (as combined with concern regarding invasive species eliminating native species) was the second ranked theme overall. Concern for endangered species was an additional theme reflecting additional interest related to native species. Finally, we found substantial interest in Human attributes, accounting for a bit less than 20% of total feedback. This includes some relatively large themes, such as appreciation of recreational amenities, and dislike of garbage.

This study is similar to previous research in southern Arizona [20]. Despite marked landscape and sociodemographic contrasts, there are numerous parallel results between the two studies. This provides evidence that what matters to people about rivers may resonate across the western US, perhaps even beyond. Yet two notable differences demonstrate the need to consider regional context. In arid southern Arizona, the top water theme was the mere presence of water in the river channel. This idea only tangentially appeared in temperate western Oregon, under the theme of appreciating river views. Second, a significant theme in Oregon was fish edibility. In Arizona it was considered interesting if fish were present at all: fish edibility does not appear as a theme. This underscores the need to consider local context when attempting to apply metrics from either study to a new location.

Our results can also be compared to river attributes appearing in the global literature. A detailed comparison is beyond our scope, but we draw preliminary insights from a limited analysis, comparing with [11], a recent review of the nonmarket valuation literature on river restoration, and [59], a meta-analysis of nonmarket river restoration values. Both studies categorize river restoration ecosystem services, and both lists bear much resemblance to our top themes. The first study [11] finds that the vast majority of studies reviewed sought to restore or protect fish populations, including many threatened or endangered fish populations. Numerous studies also focused on improving wildlife habitat, and water quality for boating. The bulk of studies included in the meta-analysis [59] valued changes in wildlife habitat (a fisheries focus is possible but is not mentioned). Second in valuation frequency was landscape esthetics, with water recreation third.

The similarity of our results with this sample of the literature is encouraging; if our results are accurate, the nonmarket valuation literature on river restoration appears to be largely on the right track. We had multiple themes of public interest regarding wildlife, especially fisheries, as well as numerous themes covering recreation, esthetics, and endangered species. However, there are also some interesting contrasts. For example, property damage from flooding, navigability of waterways, large tree vegetation, and recreational amenities are all among our top themes (Fig 3), but are not prominent in the ecosystem service categorizations of [11] or [59]. There are multiple potential explanations for this. The categories in the comparison studies are broad—two papers included in [11] do actually value tree vegetation changes [14, 47] although vegetation is not given a separate category. The river restoration focus may bias the sample towards ecological attributes rather than human features such as recreational amenities. In addition, there is some bias towards attributes that can feasibly be changed—e.g. navigability would be difficult to change for many waterways since it is highly dependent on gradient and geology. Finally, some topics such as property damage from flooding involve market impacts, and thus logically appear in a separate literature [60–62]. The flood damage example is indicative of how broad the literature review and synthesis would need to be in order to execute a detailed comparison with our results, an undertaking we must reserve for future research.

A comparison with the literature should consider metric formulations as well as river themes of public interest. A primary goal of our research was to bring the specificity of metrics to general themes such as "wildlife." Metric comparison cannot be done without close examination of measurable features explicit or implied in each individual paper. Yet one widespread type of metric formulation deserves mention here, raised by an anonymous reviewer. Often, metrics embed a comparison of river status to a "reference condition." As noted previously, our participants commonly considered what was "natural" when commenting on preferred river attributes, such as when discussing background photos. Accordingly, several of our metrics include a direct or implied comparison with natural condition, such as the invasive species metric, and the garbage metric. Whether or not a comparison with a baseline is made is a critical decision, with important philosophical consequences: there are also numerous ways to actually calculate such a comparison, leading to potential for bias in how a metric is interpreted by the public [63]. Furthermore, it should be noted that there can be challenges in establishing a reference condition, e.g. situations where human activity has left little if any evidence of natural state [64]. The issues regarding reference condition are just one example of the challenges that remain in formulating metrics, after a theme of public interest has been identified.

Our results provide strategic, detailed insights on river metrics people care about, using interactive discussion techniques best suited to isolate what really matters. It was a constant challenge for the moderator to move past commonly stated issues such as "pollution" to specific measurable attributes, requiring repeated use of follow-up and probe questions uniquely suited to qualitative methods. Our results provide high-level insight on river themes and metrics of public interest, an arena with surprisingly little exploratory research. Our provisional metrics seed future research, and also provide a reference for situations in which river metric insight is needed but funds for original research are lacking. We suggest the following three applications of our results in particular:

- Reference for design of a river monitoring program, e.g. a river ’report card.’
- Reference for how to adapt already available river information for improved public relevance.
- Reference for priority areas of river management in the public interest.

In addition to information on metrics of public interest we provide, river monitoring should also consider the costs of acquiring different kinds of information, as well as the costs and benefits of different management actions as based on river monitoring data. For example, certain kinds of information may be extremely expensive to monitor, and thus it could be optimal for these to remain poorly known despite high public interest. Information will also be a platform for management in at least some cases. Metrics feasibly influenced through management actions, and with relatively high public value per unit change, should be prioritized when designing a river monitoring plan, although these factors are not necessarily easy to evaluate a priori.

Our goal was to formulate hypotheses of public interest across the spectrum of river attributes, yet our study has limitations. With an intensively studied but only moderately sized sample, split across five segments, we cannot conduct statistical tests or claim generalizability of results to the underlying population. Complementary quantitative research is a potential avenue for further work, i.e. a mixed-methods approach [34]. Survey work to test the hypotheses of priority themes and metrics we offer in this article would be needed to precisely gauge the absolute ranking of themes and metrics for a population (e.g., "best-worst scaling" methods, [29, 30]). Follow-up research could also measure any bias resulting from different background materials provided to human subjects. Of course, any quantitative survey effort should receive dedicated qualitative pre-testing before full deployment [65].

## Conclusions

We painstakingly document publicly relevant river metrics based on detailed feedback from almost one hundred participants, recruiting both urban and rural residents of western Oregon. Our research adds further flesh and blood to the concept of Final EGS developed within environmental economics, with results oriented towards practical monitoring, modeling, and management usage for water resources professionals. We utilize qualitative research techniques of focus groups and interviews common to social science, methods preferred for eliciting human perspectives on complex questions. Of particular importance in our case, back-and-forth moderation allowed crucial follow-up opportunities to distinguish proxy issues from real concerns, e.g. interest in water clarity was not tallied as a Final EGS when the ultimate concern was actually safety of recreational water contact.

An important outcome is that meetings with diverse research participants led to highly repetitious feedback regarding what river metrics matter. We found remarkable coalescence across numerous groups and individuals, emphasizing relatively few themes overall. Furthermore, our results are similar to previous research, both prior qualitative research on river metrics for a contrasting US geography, and the global literature on nonmarket valuation. We thus speculate that there are relatively few river themes that account for the bulk of public interest, applying across diverse geographic contexts, with some allowance for regional context. We also speculate that some themes, such as those within the Biota category, would appear for studies investigating public interest in other types of ecosystem, such as oceans or forest.

The insights we provide on river metrics are important to help achieve focus in environmental study and management. In various ways, both natural scientists and social scientists work with or collect environmental data without necessarily knowing how publicly relevant the selected indices are. Thus, efforts are potentially inefficient if the ultimate goal is to manage resources in the public interest. Our results spotlight the information that effectively bridges the biophysical and social realms, and thus a potentially powerful shared set of objectives. In some cases publicly derived river metrics may imply new measurements, in other cases information may already exist or simply require changes in presentation to make the data more accessible. By providing publicly-relevant river information, we hope to make progress towards focused outcomes for river management, rather than fracturing limited resources. Our rigorously generated hypotheses regarding publicly relevant river metrics may be refined with future research, or utilized directly in appropriate circumstances. In deciding what data to collect, river monitoring should also consider the costs of acquiring different forms of information, as well as the costs and benefits of potential river management actions based on monitoring data.

## Supporting information

S1 Appendix Focus group script and validation interview specific questions. (DOCX)

S2 Appendix Willamette river background. (TIFF)

S3 Appendix Willamette river photos. (TIF)
