# Supplementary material for: River metrics by the public, for the public
Source: PLoS One. 2019 May 8;14(5):e0214986. doi: 10.1371/journal.pone.0214986 (PMC6505747; doi:10.1371/journal.pone.0214986)
Supplement: S1 Appendix — (DOCX) [file pone.0214986.s001.docx]

Focus Group Script

Part I

- Confidentiality terms, informed consent
- Warm-up & trust building
- Any questions before we begin?

Part II

- Q1: What is your familiarity with rivers and streams in this area?
- Q2: What are the first things to mind regarding rivers & streams in this area?
- Q3: Is there anything important to you about rivers & streams in this area?

Part III

- Q4: What are the first things to mind regarding the Willamette River?
- Moderator distributes river background & photos
- Q5: Is there anything important to you about the Willamette River?
- Q6: Please rank photos based on what is most important to you (emphasis on rationale & discussion).
- Q7: If you were in charge of the Willamette River how would you manage it?
- Q8: If you had to name one thing you want me to pay attention to, what is it?
- Q9: Is there anything else you’d like to add?

Member-Checking Specific Questions

- Are the categories relevant for you?
- Is the specific information relevant for you?
- What are the top 5 points of information from the 35 listed that you would emphasize?
- What 5 points of information would you drop first, if there were not enough funds to cover all 35 listed?
- Imagine you could find out more about what lives in or along a particular river. Would you be more interested in birds, fish, or mammals?
- In general, are you more interested in wildlife that can be hunted or fished, or endangered wildlife?
- Many participants have spoken about “whole ecosystem” concerns. Does the information listed cover the most important topics of river and stream ecosystems for you?
