# Supplementary figures and images for: River metrics by the public, for the public
Source: PLoS One. 2019 May 8;14(5):e0214986. doi: 10.1371/journal.pone.0214986 (PMC6505747; doi:10.1371/journal.pone.0214986)

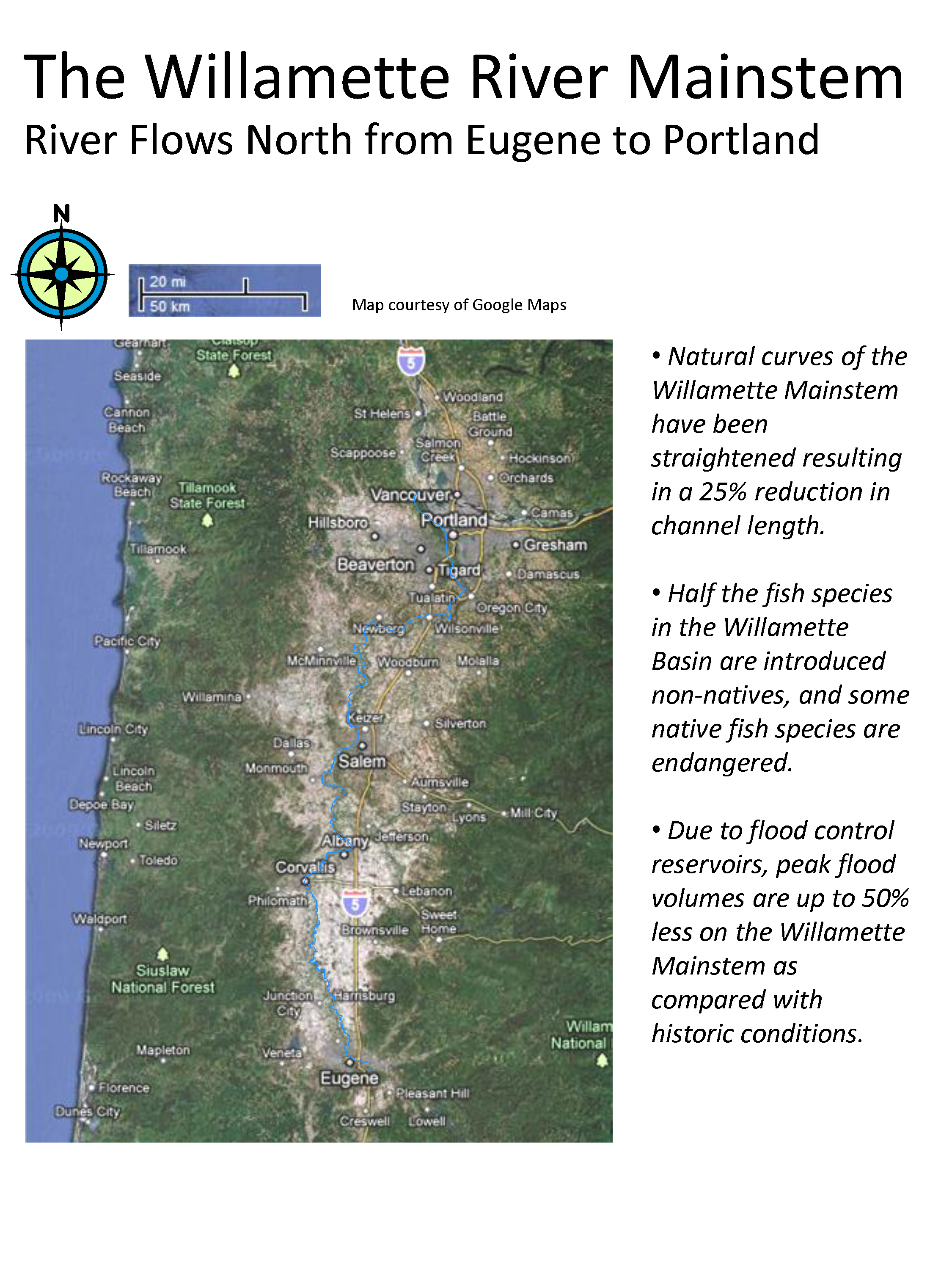

Supplement: S2 Appendix — (TIFF) [file pone.0214986.s002.tiff]

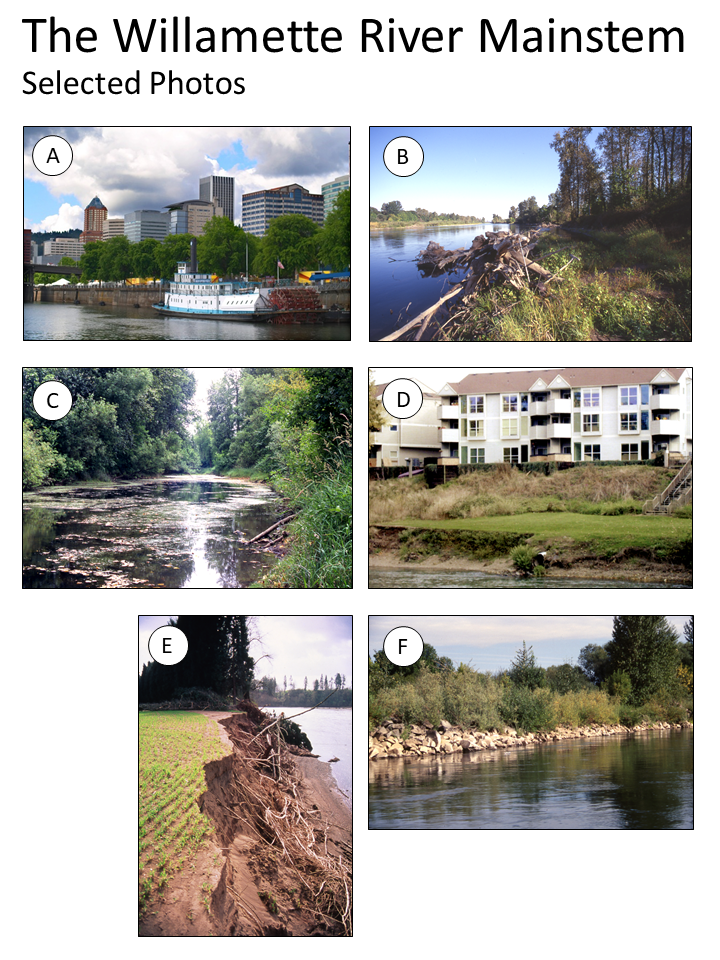

Supplement: S3 Appendix — (TIF) [file pone.0214986.s003.tif]
